# Bidirectional Crosstalk Between Hypoxia Inducible Factors and Glucocorticoid Signalling in Health and Disease

**DOI:** 10.3389/fimmu.2021.684085

**Published:** 2021-06-04

**Authors:** Tineke Vanderhaeghen, Rudi Beyaert, Claude Libert

**Affiliations:** ^1^ Centre for Inflammation Research, Flanders Institute for Biotechnology (VIB), Ghent, Belgium; ^2^ Department of Biomedical Molecular Biology, Ghent University, Ghent, Belgium

**Keywords:** glucocorticoids, glucocorticoid receptor, HIF, hypoxia, inflammation

## Abstract

Glucocorticoid-induced (GC) and hypoxia-induced transcriptional responses play an important role in tissue homeostasis and in the regulation of cellular responses to stress and inflammation. Evidence exists that there is an important crosstalk between both GC and hypoxia effects. Hypoxia is a pathophysiological condition to which cells respond quickly in order to prevent metabolic shutdown and death. The hypoxia inducible factors (HIFs) are the master regulators of oxygen homeostasis and are responsible for the ability of cells to cope with low oxygen levels. Maladaptive responses of HIFs contribute to a variety of pathological conditions including acute mountain sickness (AMS), inflammation and neonatal hypoxia-induced brain injury. Synthetic GCs which are analogous to the naturally occurring steroid hormones (cortisol in humans, corticosterone in rodents), have been used for decades as anti-inflammatory drugs for treating pathological conditions which are linked to hypoxia (i.e. asthma, ischemic injury). In this review, we investigate the crosstalk between the glucocorticoid receptor (GR), and HIFs. We discuss possible mechanisms by which GR and HIF influence one another, *in vitro* and *in vivo*, and the therapeutic effects of GCs on HIF-mediated diseases.

## Glucocorticoids and the Glucocorticoid Receptor: A General Introduction

Glucocorticoids (GCs; corticosterone in rodents, cortisol in humans) are important steroid hormones which play a role in several fundamental physiological processes such as lipolysis (1) and gluconeogenesis (2), inflammation (3), development (4) and reproduction (5), growth (6), mood and cognition (7, 8), and cardiovascular function (9). They are mainly synthesized in the cortex of the adrenal glands by enzymatic processing of cholesterol (10). Extra-adrenal GC production in the thymus, brain, vasculature, and epithelial barriers has also been observed, where GCs primarily regulate local immune responses (11).

### The Biosynthesis of GCs, Their Regulation, and Biological Activity

**Figure 1:**
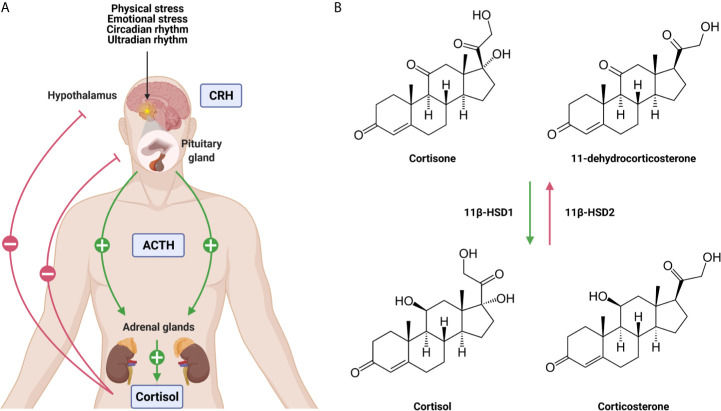
The hypothalamic-pituitary-adrenal (HPA) axis **(A)** and the conversion of glucocorticoids (GCs) **(B)**. **(A)** Under unstressed, homeostatic conditions GCs are released from the adrenal glands in the blood stream in a circadian and ultradian rhythm. Upon physiological and emotional stress, this release is increased. Stimulation of the HPA axis induces the secretion of corticotrophin releasing hormone (CRH) by the hypothalamus which stimulates the release of adrenocorticotrophic hormone (ACTH) by the pituitary. This will lead to the production of GCs by the adrenal glands and the secretion into the blood stream. Blood GC levels regulate the hypothalamus and the pituitary *via* a negative feedback loop. **(B)** The lipophilic GCs diffuse freely through the plasma membrane. The bioavailability of the GCs is regulated by 11β-HSD1 and 11β-HSD2 which are responsible for the conversion of inactive cortisone/11-dehydrocortisone into active cortisol/corticosterone and *vice versa*. Figures created with Biorender.

The adrenal GC production is regulated by the hypothalamic-pituitary-adrenal (HPA) axis (**Figure 1A**). Under homeostatic, unstressed conditions, the adrenal glands release GCs into the bloodstream in a circadian and ultradian rhythm characterized by peak levels in the morning in humans and in the late afternoon/early night in nocturnal animals such as mice. Upon physiological (e.g. activated immune response) and emotional stress, the activity of the HPA axis is increased. Furthermore, the production of cytokines during inflammation also activates the HPA axis which forms an important regulatory process. GCs will limit the production of most cytokines to maintain homeostasis and guarantee the survival of the host to a life-threatening impact of excessive inflammation, which can be described as a tolerance-like mechanism (12).

When the HPA axis is stimulated, the hypothalamus secretes corticotropin-releasing hormone (CRH) which subsequently induces the secretion of adrenocorticotropic hormone (ACTH) by the anterior pituitary in the bloodstream. ACTH will then bind to its receptor and stimulate the adrenal glands to synthesize and secrete GCs into circulation (13). Blood GC levels are regulated by a negative feedback loop, whereby the HPA axis is inhibited at different levels by GCs both in a genomic and non-genomic way (14). The total amount of GCs in circulation is controlled by the adrenal production, but extracellular binding proteins and intracellular enzymes regulate local GC activity. In plasma, ~90% of circulating GCs are bound by corticosteroid-binding globulin (CBG) and albumin, thereby leaving only a limited amount of circulating GCs in a free, biological active form (15). At sites of infection, proteases such as neutrophil elastases target CBG, causing the local release of bound GCs (16). Once the lipophilic GCs are released into the bloodstream, they diffuse through cell membranes to bind cytosolic glucocorticoid receptor (GR). This receptor is ubiquitously and constitutively expressed throughout the body, but exerts tissue-specific and cellular effects (17). The bioavailability of GCs in the cytoplasm is determined by the balance between active and inactive forms of GCs. Within cells, 11β-hydroxysteroid dehydrogenase (11β-HSD1/2) enzymes are responsible for the conversion of inactive cortisone/11-dehydrocortisone into active cortisol/corticosterone, and *vice versa* (**Figure 1B**). Inflammatory cytokines are able to regulate the expression of 11β-HSD1/2 enzymes, thereby modulating GC activity locally (18).

### The GR Protein Structure

**Figure 2:**
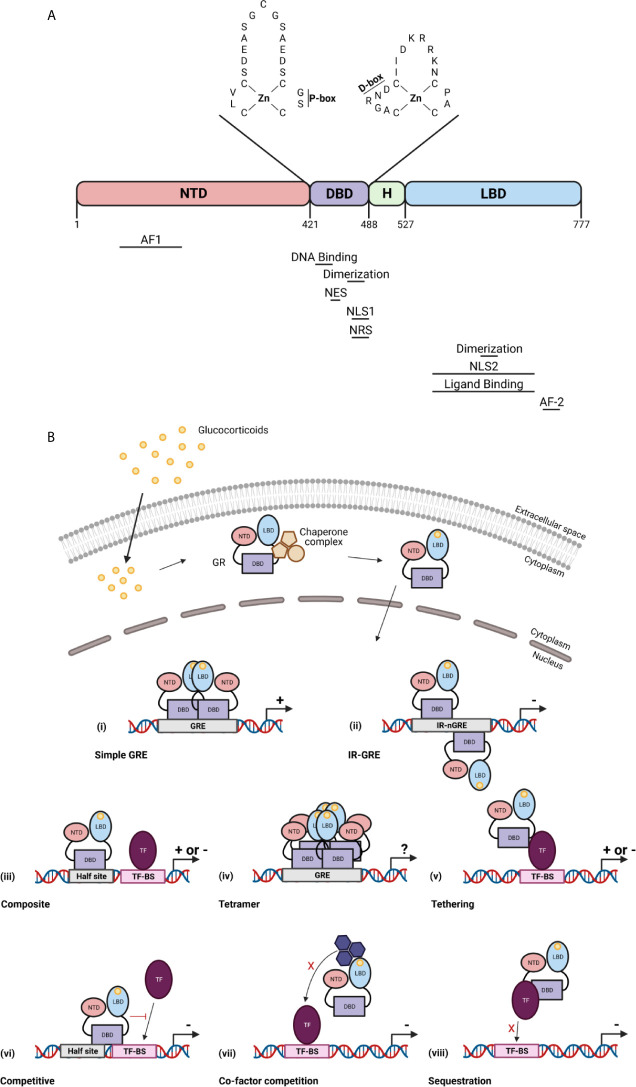
The glucocorticoid receptor (GR) protein **(A)** and its activation and function **(B)**. **(A)** The structure of the GR protein containing an N-terminal domain (NTD), a DNA-binding domain (DBD), a hinge region (H), and a ligand-binding domain (LBD). The DBD contains two zinc fingers important for specific GR DNA binding (P box) and GR dimerization (D box). Regions important for the GR function are indicated below the protein structure. AF, activation function; NLS, nuclear localization signal; NES, nuclear export signal; NRS, nuclear retention signal. **(B)** GCs diffuse through the cell membrane and bind the GR in the cytoplasm. This causes a change in the chaperone complex followed by its release and the translocation of the GR to the nucleus where it induces transactivation (+) or transrepression (–) of gene transcription as a GR monomer or a GR homodimer. The GR transactivate gene expression by binding to glucocorticoid receptor element (GRE) as a dimer (i), or can transrepress gene expression by binding to inverted repeat negative GREs (ii). The GR further transrepresses the expression of genes *via* binding to composite elements (iii), by tethering (v), by competing for DNA binding sites (BS) (vi), by competing for co-factors with other TFs (vii) or by sequestrating TFs (viii). GR can also function as a tetramer (iv), but its function is unknown. Figures created with Biorender.

The GR is a member of the nuclear receptor superfamily of transcription factors (TFs). It is a 97 kDa protein encoded by the *NR3C1/Nr3c1* gene (chromosome 5 in human, chromosome 18 in mouse). The GR protein contains an N-terminal domain (NTD), a DNA-binding domain (DBD), a hinge region, and a C-terminal ligand-binding domain (LBD) (19). In the NTD, the ligand independent activation function (AF1) is present which is responsible for the binding of co-factors, chromatin modulators, and the transcription machinery (20). The DBD contains two subdomains each containing a zinc finger important for specific GR DNA binding (proximal box) and GR dimerization (distal box) (21). The hinge region provides flexibility between the DBD and the LBD and acts as a regulatory interface. The LBD contains a ligand binding pocket which is composed of 12 α-helices and 4 β-sheets, and the ligand-dependent AF-2 domain. GR dimerization is also provided by a dimerization interphase in the LBD (22). Further, nuclear localization (NLS), nuclear export (NES), and nuclear retention (NRS) signals have been identified in the GR and are required for the subcellular traffic of GR (**Figure 2A**) (23).

### GR Signalling

In the absence of bioactive ligand, the GR resides in the cytoplasm in a multiprotein chaperone complex. This chaperone complex is critical for the conformation of GR, in an ATP dependent manner, in which the ligand-binding cleft of GR is opened to allow GC binding with high affinity. Furthermore, the chaperone complex is important for nuclear translocation and activation of the GR. Upon ligand binding, the GR undergoes conformational changes leading to dissociation of the chaperone complex and translocation to the nucleus (24). Once in the nucleus, the GR transcriptionally activates or represses gene expression as a GR monomer or a GR dimer (or even a tetramer) usually *via* direct interaction with specific DNA sequences (25). In most tissues and in the presence of endogenous GCs during homeostasis, GR most frequently binds to DNA as a monomer and interacts with DNA *via* binding to GC response element (GRE) half-sites. When a binding site for another TF is in close proximity of the GRE half-site, both elements are able to act as a composite site where the GR monomer and the other TF might interact in a positive or negative manner (26). Two GR monomers can also bind to DNA *via* interaction with inverted negative GREs and repress gene expression by recruitment of corepressors (27). When exogenous GCs like dexamethasone (DEX) and prednisolone are administered, the binding of the GR dimer to GREs is favoured at the cost of the GR monomer. Of course, GR homodimers are also formed under certain pathophysiological conditions, e.g. when GCs rise very high in the blood. Binding of GR homodimers induces transcriptional activation of genes, which indicates that the GR monomer is the most important for physiological functions whereas the GR dimer is crucial for the pharmacological and stress functions (28). GR can also interact with specific genome regions indirectly *via* tethering with other TFs such as nuclear factor κB (NF-κB) and AP-1 (29). Additionally, GR is able to compete with other TFs for overlapping DNA binding sequences (30) of for the binding of co-factors (31, 32). Finally, GR can sequester other TFs to prevent them of binding to the DNA (**Figure 2B**) (33). Next to genomic effects, GR can also induce non-genomic effects which are not dependent on transcriptional activities or protein synthesis (34).

## The Physiological Regulation and Structure of Hypoxia-Inducible Factors

The discovery of how cells are able to sense and respond to low oxygen levels, known as hypoxia, has been rewarded in 2019 with the Nobel Prize in Physiology or Medicine, for Drs. Ratcliffe, Kaelin and Semenza. The regulation of oxygen homeostasis and the access to an adequate oxygen supply is crucial for the survival of all aerobic organisms, including humans. During oxygen deprivation induced by low levels of haemoglobin or insufficient blood flow to specific organs, cells modulate their protein activity or change their transcriptional and post-transcriptional organisations (35). Cells will activate multiple genes involved in diverse biological processes such as cell survival and proliferation (36), glucose metabolism (37), and angiogenesis (38).

### Oxygen Sensing and the Importance of HIF Proteins

The human body senses oxygen levels by different mechanisms. First, the carotid body located in the carotid artery senses oxygen and carbon dioxide levels in the blood. When the carotid body detects a decrease in blood oxygen levels, it transduces a signal to stimulate breathing, thereby increasing the acquisition of oxygen from the air (39). Second, an unexpected and novel role for the mouse olfactory system has been revealed as a peripheral oxygen-sensing system that enables mice to rapidly assess the oxygen level in the environment before the arterial blood becomes hypoxic (40). Also, oxygen-dependent enzymes such as 2-oxoglutarate-dependent [2-OG, also known as α-ketoglutarate (α-KG)] oxygenases require oxygen for their activity. When oxygen levels decrease, their activity is inhibited. Prolyl hydroxylases (PHDs) are the best-characterized 2-OG-dependent oxygenases which negatively regulate hypoxia inducible factors (HIFs) (41), and thus operate as an “oxygen sensor” system. Additionally, mitochondria are also considered as a site of cellular oxygen sensing since they are responsible for the majority of oxygen consumption within cells. Oxygen is used at the terminal complex in the electron transport chain (ETC), where oxygen serves as the acceptor of protons, which have been stripped from metabolites in the ETC, forming H_2_O. The production of mitochondrial reactive oxygen species (ROS) signals upon hypoxia was firstly demonstrated by Chandel et al. They have shown that HIF1α is stabilized upon hypoxia by the generation of ROS by the mitochondrial ETC (42), and more specifically, the ROS generated by complex III (43). A possible explanation how mitochondrial ROS stabilize HIF proteins is by inhibiting PHDs and Factor inhibiting HIF1 (FIH). Hydroxylases can be modified post-translationally by redox signals. Since PHD2 is able to interact with other proteins (44), ROS signals can influence PHD2 activity by changing these protein-protein interactions. Another possibility is the oxidation of cysteine residues or attacking iron (Fe^2+^) atoms (45, 46), which are important for PHD and FIH function, by ROS leading to the inactivation of these oxygen-dependent enzymes.

**Figure 3:**
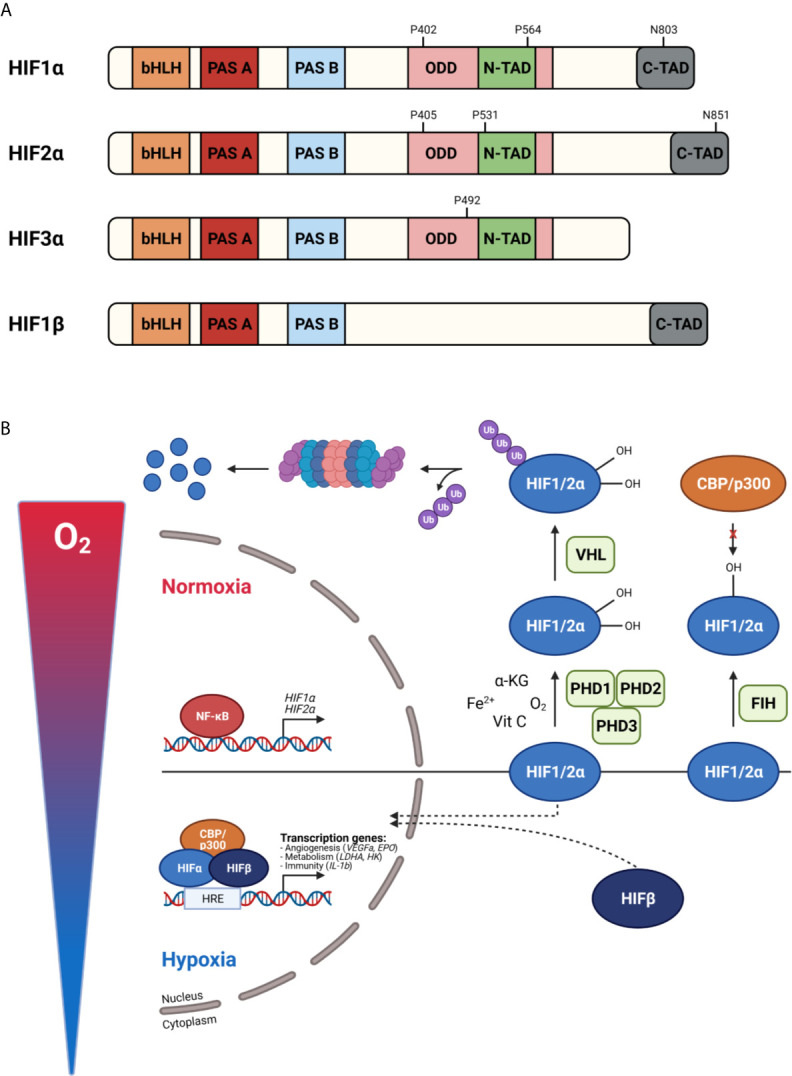
Protein structure of hypoxia inducible factors (HIFs) **(A)** and their activity under normoxic and hypoxic conditions **(B)**. **(A)** HIF proteins are basic helix-loop-helix Per-Arnt-Sim (bHLH-PAS) transcription factors build up by an α-subunit and β-subunit. HIF1α consist of a bHLH and PAS domain, necessary for DNA binding and homodimerization, respectively. Further, an oxygen-dependent domain (ODDD) and two transactivation domains (N-TAD and C-TAD) are present. The stability and target gene specificity are determined by the N-TAD which overlaps with the ODDD. The C-TAD regulates the interaction with co-activators thereby activating gene transcription. HIF1α and HIF2α differ within their transactivation domain, while HIF3α is known as the dominant-negative regulator. Due to the absence of the ODDD, HIF1β is constitutively expressed. **(B)** In normoxia, prolyl hydroxylases (PHDs) hydroxylate proline (P) residues in the ODDD leading to the binding of the von Hippel-Lindau protein (pVHL) followed by ubiquitination and degradation by the 26S proteasome. The factor inhibiting HIF1 (FIH) hydroxylates asparagine (N) residues in the C-TAD, preventing the interaction of the C-TAD with co-activators thereby inhibiting gene transcription. When oxygen levels decrease, PHDs are inactivated, the HIFα subunits are stabilized, dimerize with HIFβ and translocate to the nucleus. After CBP/p300 are recruited, gene transcription is induced by binding to hypoxia-responsive elements (HREs). Figures created with Biorender.

The master regulators involved in oxygen homeostasis and important for development, physiology, and disease are HIFs. They are members of the basic helix-loop-helix Per-Arnt-Sim (bHLH-PAS) transcription factor superfamily. HIFs are heterodimers containing an α- and β-subunit. Three α-subunits are found in mammals, namely HIF1α, HIF2α, and HIF3α, which are oxygen-sensitive as they accumulate in hypoxia. The β-subunit, also known as the aryl hydrocarbon receptor nuclear translocator (ARNT), is constitutively expressed and not affected by hypoxia. HIF1α and HIF1β contain an N-terminal bHLH and PAS domain, responsible for DNA binding and heterodimerization, respectively, and C-terminal transactivation domains (N-TAD and C-TAD). N-TAD is mandatory for target gene specificity and the stability, while C-TAD interacts with co-activators e.g. p300 and CREB binding protein (CBP) required for full HIF activity and regulation of HIF target gene expression. The oxygen sensitivity of HIFα is conferred by an internal oxygen-dependent degradation domain (ODDD). Besides these domains, two nuclear localization signals (N-NLS and C-NLS) are present, which direct the HIF protein to the nucleus. HIF2α exhibits high structural similarity with HIF1α, but they differ in their transactivation domains leading to differences in target gene specificities. HIF1α is ubiquitously expressed, while HIF2α expression is more cell type specific like hepatocytes, adipocytes, endothelial cells, cardiomyocytes, interstitial cells, kidney glomeruli and neurons (47–52). HIF3α is the dominant-negative regulator of the HIF pathway. Due to the absence of the ODDD, HIF1β is constitutively expressed in an oxygen-independent manner (**Figure 3A**).

### HIF Protein Regulation

In physiological oxygen conditions, HIFα proteins are constantly produced and degraded by the 26S proteasome. When oxygen is present, PHDs hydroxylate proline (Pro) residues present in the ODDD (Pro 402 and 564 for HIF1α, Pro 405 and 531 for HIF2α) inducing an ubiquitination reaction by the E3 ubiquitin ligase Von Hippel–Lindau protein (pVHL) followed by 26S proteasome-mediated degradation. The activity of PHDs relies on the presence of oxygen, Fe^2+^, α-KG and ascorbic acid (also known as vitamin C). Each PHD has a different affinity for a certain HIF. HIF1α is mainly regulated by PHD2, while PHD1 and PHD3 are the master regulators of HIF2α (53–55). Mouse studies have reported that PHD2 deficiency causes defects in the developing heart and placenta leading to embryonic lethality between E12.5 and E14.5 (56, 57). Deletion of PHD1 and PHD3 is not lethal and only has tissue specific effects, because of their role in cellular metabolism in skeletal muscle and blood pressure in the central nervous system, respectively (58, 59). Hydroxylation of asparagine (Asn) residues in the C-TAD (Asn 803 for HIF1α and Asn 851 for HIF2α) by FIH prevents the interaction of C-TAD with co-activators causing the inhibition of the transcriptional activity of HIFα. Under hypoxic conditions, or in the absence of the cofactors Fe^2+^, α-KG or vitamins, PHDs are inactivated and HIFα hydroxylation is inhibited. The availability of HIFα can also be regulated at a transcriptional level *via* a crosstalk with other signalling pathways. For example, NF-κB is able to bind the HIF1α promotor and induces its transcription (60). Once the α-subunits are stabilized, they dimerize with HIF1β and translocate to the nucleus. After co-factor recruitment, the heterodimeric complex binds to hypoxia responsive elements (HREs) containing the core sequence RCGTG (R: A/G) leading to the activation of target genes (**Figure 3B**).

## Bidirectional Crosstalk Between HIF and GR

### The Direct Effect of Hypoxia/HIF on GR Function and *Vice Versa*

**Figure 4:**
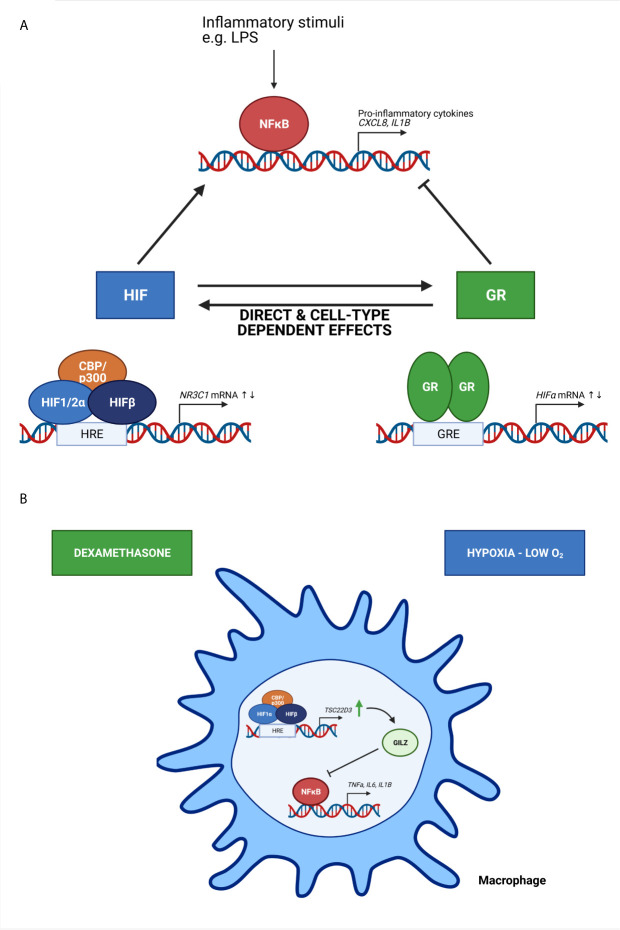
The crosstalk between HIF and GR and its effect on NF-κB activity **(A)** and the effect of oxygen availability on immune cells **(B)**. **(A)** Based on *in vitro* studies, HIF and GR have direct and cell-type dependent effects on each other, thereby increasing or repressing the transcription of *HIF* mRNA and *N3RC1* mRNA genes. Inflammatory stimuli like lipopolysaccharide (LPS) induce NF-κB mediated transcription of genes encoding pro-inflammatory cytokines. HIF is known to stimulate this NF-κB mediated gene transcription, while GR will repress this. **(B)** Upon hypoxic conditions, the expression of GC-induced leucine zipper (GILZ) encoded by the *TSC22D3* gene is upregulated, thereby inhibiting the expression of pro-inflammatory cytokines mediated by NF-κB. When macrophages are pre-treated with dexamethasone, a synthetic GC, the effects are amplified. Figures created with Biorender.

The presence of a crosstalk between hypoxia-dependent signalling pathways and GCs and their receptor have been described in several *in vitro* studies (**Figure 4**). Kodama et al. have been the first to provide evidence of an interaction between HIF and GR. They have identified that hypoxia-dependent gene expression and HRE activity in HeLa cells is enhanced by ligand-dependent activation of GR after DEX stimulation. Furthermore, they show that the LBD is essential for the induction of HRE-Luc activity in HeLa cells upon DEX stimulation, since the HRE-Luc activity is not altered when HeLa cells are transfected with a GR plasmid lacking the NTD or in the presence of the point mutation A458T causing impaired GR dimerization. Although they have no direct evidence of a clear protein-protein interaction between the GR LBD and HIF1, colocalization of GR and HIF1 in distinct compartments of the nucleus are shown (61). Subsequently, a functional role for hypoxia and HIF1α in the regulation of GR mRNA and protein expression and the associated increased GC activity has been shown by Leonard et al. in human proximal tubular epithelial cells (HK-2 cells), indicating that there is an obvious crosstalk between HIF and GR. The upregulation of GR might occur through the binding of HIF1α to one or more HRE sites in the NR3C1 promotor, thereby enhancing GR transcription (62). These results were confirmed in another study where GR expression is upregulated in mouse pituitary AtT-20 cells in hypoxic conditions by increased HIF1α mRNA and protein expression levels (63).

In contrast to the previous studies, Wagner et al. demonstrate that the effect of hypoxia on human hepatoma HepG2 cells in combination with DEX stimulation leads to reduced HIF1α DNA binding activity and HRE activity in a dose-dependent manner. Also, the expression of endogenous HIF1α target genes is decreased. This indicates that DEX attenuates HIF1α activity in a GR-dependent manner, since these effects are not present in a GR deficient human hepatoma Hep3B cell line and are restored upon transient expression of GR. They also report an unusual distribution of HIF1α in the cytoplasm, which suggests a problem with nuclear translocation of HIF1α (64).

### The Consequences of Hypoxia on the Anti-Inflammatory Actions of GCs

Next to the direct effect of hypoxia/HIF on GR and *vice versa*, several *in vitro* studies describe the effect of hypoxia on the anti-inflammatory actions of synthetic GCs. Hypoxia plays a key role in chronic lung diseases e.g. asthma and chronic obstructive pulmonary disease (COPD). The alveolar epithelial cells are directly exposed to changes in oxygen pressure in the arterial blood and to inhaled steroids (65). The exposure of A549 cells (immortalized human alveolar epithelial cells) to hypoxic conditions causes a downregulation of both GR mRNA and protein levels (66) and inhibits nuclear translocation of GR (67), which is in contrast with the results of Leonard et al. (62). This suggests that hypoxia has cell-type-specific effects on GR mRNA and protein levels (68). In the presence of normal oxygen levels, DEX inhibits the production of IL-8 by A549 cells when stimulated with lipopolysaccharide (LPS). However, hypoxia significantly attenuates the DEX-mediated inhibition of IL-8 production in these cells (66). Charron et al. also demonstrate a reduced anti-inflammatory effect of DEX: stimulation of A549 cells with the pro-inflammatory cytokine IL-1β leads to increased CXCL8 production, which can be repressed by DEX, but in hypoxia the DEX response is less effective (**Figure 4A**). They show that this reduced DEX effect might be linked to the binding of HIF1α to HREs present in the promotor of histone deacetylase 2 (HDAC2), which is normally recruited by an activated GR to repress NF-κB mediated transcription in airway epithelial cells. However, binding of HIF1α to the promotor leads to reduced *HDAC2* transcription and less suppression of NF-κB mediated transcription of genes with known pro-inflammatory functions (69).

### Immune Cells and the Role of Oxygen Availability

Oxygen availability is also important for the functional behaviour of immune cells. Hypoxia is able to activate monocytes, macrophages and dendritic cells by altering their gene expression and cytokine secretion (70–73). GC-induced leucine zipper (GILZ) is encoded by the *TSC22D3* gene, which is known as a DEX-inducible gene and is highly expressed in cells of the immune system (74). GILZ is able to inhibit the activation of macrophages and the production of pro-inflammatory cytokines and inflammatory mediators upon exposure to LPS (75). The exposure of macrophages to hypoxic conditions significantly upregulates GILZ mRNA expression, thereby inhibiting the production and secretion of pro-inflammatory cytokines IL-1β and IL-6. When pre-treated with DEX these effects are amplified. These results indicate that GILZ prevents the overactivation of immune cells and overproduction of pro-inflammatory cytokines in hypoxic microenvironments and might be involved in systemic adaptation to hypoxia (**Figure 4B**) (76). In A549 cells, hypoxia induces the expression COX-2 *via* NF-κB, which is suppressed by DEX. The inhibitory effects of DEX on hypoxia-induced COX-2 is mediated by GILZ *via* a physical interaction between GILZ and HIF1α (77).

Macrophages are known to be key players in inflammatory diseases. Next to their protective immunological function, they also induce the production of pro-angiogenic cytokines and growth factors such as vascular endothelial growth factor (VEGF-A) and basic fibroblast growth factor (FGF-2) thereby promoting angiogenesis (78). Upon hypoxia, the polarization of macrophages is promoted towards the activated, anti-inflammatory M2 phenotype, and not to the pro-inflammatory M1 phenotype. This causes an increased the expression of VEGF and decreased the release of pro-inflammatory cytokines, thereby regulating macrophage functions including tumour promotion in cancer (79). It is known that GCs are angiostatic and are used to tread angiogenesis-related diseases such as solid tumours. They regulate angiogenesis *via* the suppression of proliferation, migration and sprouting in endothelial cells and by reducing the expression or secretion of cytokines and proteins involved in angiogenesis (80). Since both hypoxia and GCs are involved in angiogenesis and influence the macrophage function e.g. by inducing GILZ expression, it is possible that the hypoxic tumour environment in combination with GC treatment also causes the upregulation of GILZ expression and thereby preventing overactivation of immune cells. However, further investigation is necessary to elucidate the connection between hypoxia, GCs and GILZ expression and macrophage polarization. Must be added at the end of this paragraph: An overview of the most essential key studies concerning the bidirectional crosstalk between GCs and HIF can be found in **Table 1**.

**Table 1:** Overview of key studies concerning the bidirectional crosstalk between GC signalling and HIF mediated pathways.

	Purpose	Results	References
*In vitro*	DEX effect on HeLa cells under hypoxic conditions	Induction of hypoxia-dependent gene expression	(61)
		Increased HRE-luciferase activity	
		The LBD of GR is necessary for HRE-luciferase activity	
	Exposure of HK-2 cells or AtT-20 cells to hypoxia	Upregulation of GR mRNA and protein levels due to binding of HIF1α to HREs present in the *NR3C1* promotor	(62, 63)
	The effect of GCs on HIF1α function (HepG2 cells)	Attenuation of HIF1α activity upon hypoxia and DEX stimulation as a results of reduced DNA binding and HRE activity associated with problems with HIF1α nuclear translocation	(64)
	Characterization of the hypoxic effect on GR levels and its anti-inflammatory actions in A549 cells	Hypoxia causes a time-dependent downregulation of GR mRNA and protein levels and inhibits GR nuclear translocation	(66, 67, 69)
		The anti-inflammatory effect of DEX is attenuated when A549 cells are exposed to hypoxia and stimulated with LPS or IL-1β	
	Effect of chemical hypoxia (CoCl_2_) and/or DEX on RAW264.7 cells	GILZ is upregulated by hypoxia and is further increased upon DEX stimulation to prevent overactivation of immune cells (macrophages) and overproduction of pro-inflammatory cytokines (inhibition of IL-1β and IL-6 production)	(76)
*In vivo*	How is hypoxia affecting the endogenous GC pathway and vice versa in zebrafish larvae?	GCs stabilize HIF *via* pVHL degradation HIF represses GR activity and the GR response to exogenous GCs (e.g. BME) in *vhl^-/-^* zebrafish larvae	(81, 82)
		Cortisol levels are reduced by HIF due to repression of POMC expression and intracellular blocking the transcriptional activity of GR	
GCs in AMS	Prophylactic effect of GCs when ascending to high altitude	Administration of GCs (DEX and prednisolone) prior to ascending to high altitude reduces the symptoms of AMS (suppresses inflammatory pathways, reduces vascular permeability and vasoconstriction, improves arterial oxygenation and induces a better antioxidant-oxidant balance)	(83–88)
Perinatal hypoxia and GCs	Effect of GCs during perinatal hypoxia	Neonatal hypoxia leads to the activation of the HPA axis in the neonates and causes higher GC levels	(89, 90)
		GCs can have neuroprotective effects on neonatal HI-induced brain damage	(91–93)
		Ideal timing, dose and duration of GCs is necessary to prevent neurotoxic effects	(94, 95)

### The *In Vivo* Crosstalk Between HIF and GR and Its Role in Inflammation

From these *in vitro* studies, a dynamic interaction between oxygen concentration and GR function mediated through HIF1α has become clear (**Figure 4A**). Also *in vivo* studies using zebrafish have identified new activators of the HIF signalling pathway in the liver e.g. betamethasone (BME) and DEX, synthetic GR agonists. These GCs activate HRE reporters in a GR dependent manner (81), although *via* a non-transcriptional route since these HIF transcriptional responses were still present when the GR DBD harbours a point mutation (R443C; R484C in mouse and R477C in human), a missense mutation in the DBD thereby largely eliminating the transcriptional activity of GR (96). They suggest a mechanism by which GCs stabilize HIF proteins *via* the degradation of pVHL (81). Upregulation of the HIF signalling in *vhl* ^-/-^ zebrafish represses the GR activity and dampens its responsiveness to BME. Also endogenous cortisol levels were reduced, most likely due to HIF-mediated downregulation of POMC activity. The inhibition of the HIF pathway leads to a significant increase in both GR activity and cortisol levels (97). These results are confirmed by Marchi et al. They have also demonstrated the repression of GR activity and its reduced responsiveness to exogenous GCs when HIF protein levels are upregulated in *vhl^-/-^* zebrafish. Furthermore, cortisol levels were also reduced suggesting that HIF signalling can act both at the level of the hypothalamus by inhibiting *Pomc* expression and intracellularly by blocking the transcriptional activity of GR itself (82). A recent study by Watts et al. propose HIF1α as a vital regulator of steroidogenesis by controlling the expression of enzymes involved in steroid production (98).

In the presence of inflammation, an important role for the GR-HIF1α signalling pathway has been implicated by Lu et al. (99). Immune mediated hepatic injury (IMH) caused by LPS leads to a decrease in GR protein levels in myeloid derived suppressor cells (MDSCs), which are known to be negative regulators of the immune response (100). However, when these MDSCs are treated with DEX, GR expression is restored followed by an ameliorated mortality and reduced inflammatory insults in IMH. This implies that the GR signalling pathway in MDSCs is a potential therapeutic target in treating innate immune cell-mediated hepatic injury. They also elucidate the suppression of HIF1α and HIF1α mediated glycolysis in MDSCs thereby promoting the immune suppressive activity in MDSCs (99). Pulmonary arterial hypertension (PAH) is a progressive and life-threatening disease with poor prognosis characterized by pulmonary vasoconstriction and increased pulmonary vascular resistance leading to right ventricular failure, fluid overload and eventually death (101). The infiltration of inflammatory cells e.g. T cells, B cells, macrophages and dendritic cells is typically present in the pulmonary vascular lesions of PAH patients (102). A critical role for serum GC regulated kinase 1 (SGK-1) in the pro-inflammatory response in hypoxia-induced PAH is demonstrated. In the absence of SGK-1, the hypoxia-induced PAH development and pulmonary arterial remodelling is ameliorated, and the production of pro-inflammatory cytokines such as TNFα and IL-6 are inhibited. This suggest that SGK-1 plays a critical role in PAH development and might be a potential therapeutic target (103).

Based on these *in vitro* and *in vivo* studies, a bidirectional crosstalk between GR and HIF is present. However, more studies using animal models will be necessary to elucidate this interaction in more detail and how important this crosstalk is in inflammatory disease models. Further understanding of the GR-HIF crosstalk might lead to the development of alternative therapeutic strategies.

## Impact of GCs on Acute Mountain Sickness and the Importance of NF-κB

Hypoxia and inflammation are unequivocally linked (104). Just as hypoxia causes inflammation by stimulating NF-κB gene transcription and the production of pro-inflammatory cytokines, inflamed tissue can also become hypoxic (105). The increased demand for oxygen and decreased oxygen supply are the main reasons why tissue becomes hypoxic during inflammation. The metabolic activity following an immunogenic insult is enlarged in the inflamed tissue and requires an increased synthesis of inflammatory cytokines and enzymes leading to a higher oxygen demand (106). Additionally, the influx of immune cells at the site of inflammation leads to more oxygen consumption and cellular hypoxia (105). Another cause of hypoxia during inflammation is disrupted oxygen delivery due to vascular dysfunction (107). Therefore, both an increased oxygen consumption and decreased oxygen delivery finally lead to tissue hypoxia during inflammation.

### The Prophylactic Effect of Synthetic GCs When Ascending to High Altitude

**Figure 5:**
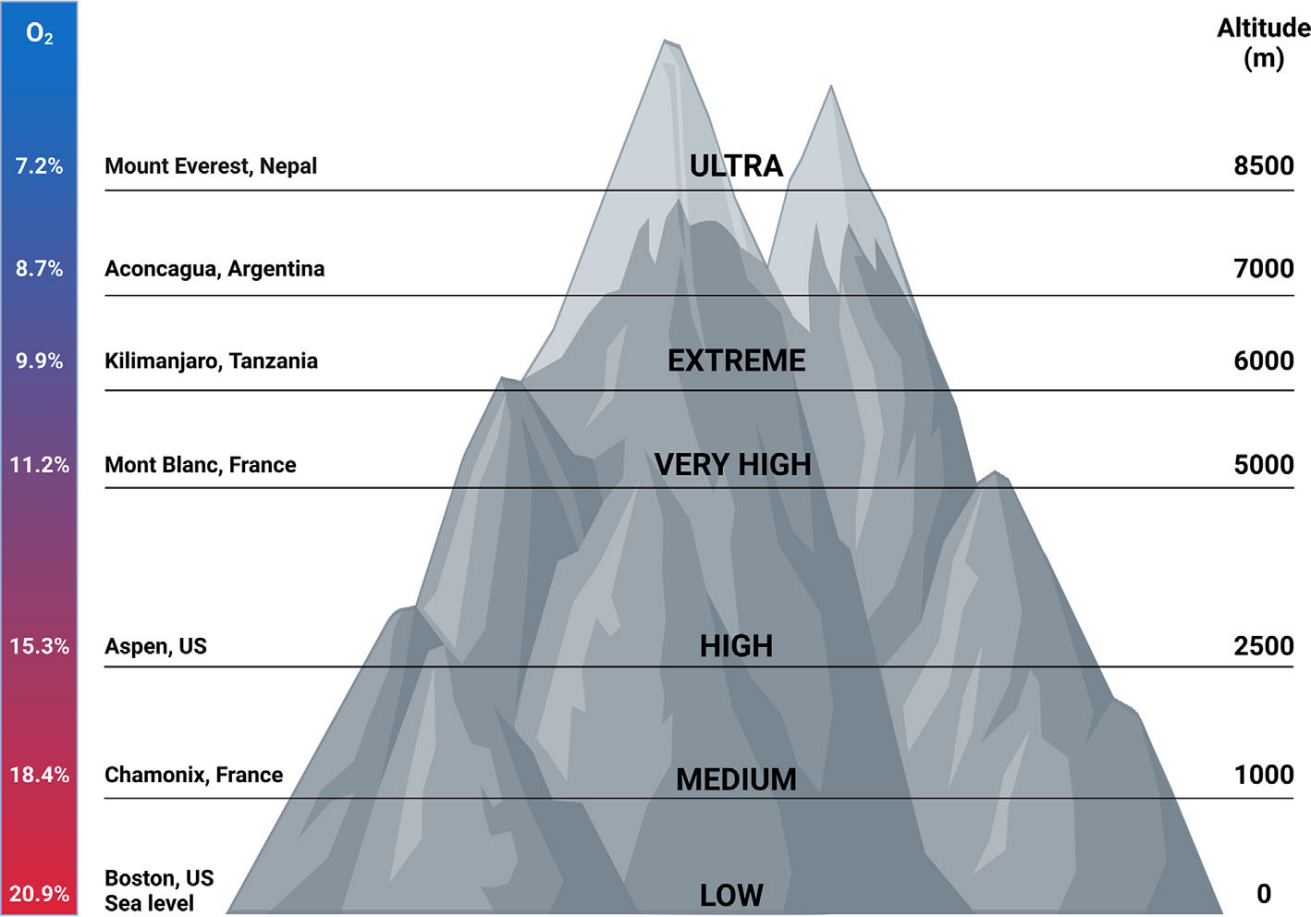
Correlation between altitude and oxygen concentrations. Millions of people live permanently at high altitude. Also travelling to regions of high altitudes has gained interest throughout the years. In this figure, oxygen concentrations correlated with certain altitudes are depicted. In addition, the grade of hypoxia present at certain places worldwide are shown. Figures created with Biorender.

Millions of people live permanently at high altitude. The inhabitants living at the highest altitude are found in La Rinconada in the Southern of Peru, where the altitude is 5100 m and only 7000 people live there. However Cerro de Pasco in Peru with an altitude of 4300 m has up to 75000 inhabitants and more than 1 million people reside at El Alto in Bolivia (altitude 4100 m) (**Figure 5**) (108). In the recent years, the interest in carrying out activities at high altitudes has increased. People travel to regions of high altitudes (i.e. higher than 2500 m) for work, permanent residence, sports and tourism. However, when ascending to high altitude, people are exposed to hypobaric hypoxia. Dependent on the length and time spent at high altitude, this can result in the development of high altitude illnesses encompassing pulmonary and cerebral syndromes which occur in non-acclimatized individuals shortly after rapid ascent (109, 110). Acute mountain sickness (AMS) is the most common syndrome and is characterized by headache and can also be accompanied by nausea, loss of appetite, disturbed sleep, fatigue and dizziness within 12h after ascent. A grading system that is used for the diagnosis of AMS is the Lake Louis self-assessment questionnaire (LLS) (111–113). AMS is a phenomenon of systemic hypoxia, vascular leakage and increased levels of circulating pro-inflammatory cytokines which can lead to the development of high altitude pulmonary edema (HAPE) or high altitude cerebral edema (HACE) and are a potentially fatal consequence (109, 114).

Several clinical trials to prevent AMS have been performed in which acetazolamide and synthetic GCs such as DEX, budesonide and prednisolone were used. Acetazolamide has been the first drug used in a prophylactic manner, however symptoms were only partly controlled and the drug caused some undesirable side effects (83). Prophylactic treatment with GCs is able to reduce the symptoms of AMS when administered prior to ascending to high altitude (83–88). Both DEX and prednisolone are able to suppress inflammatory pathways (115), reduce vascular permeability and vasoconstriction (116), improve arterial oxygenation (88) and induce a more favourable antioxidant-oxidant balance (117). Budesonide, in contrast to DEX and prednisolone, is not recommended for the prevention of AMS, although it is able to reduce the heart rate and increases the oxygen saturation (118, 119). Next to synthetic GCs, clinical trials using non-steroidal anti-inflammatory drugs (NSAIDs) e.g. ibuprofen against AMS suggest that NSAIDs might be a safe and effective alternative medicine in the prevention of AMS (120). Further, large randomized controlled clinical trials are necessary to look in more detail to the prophylactic effect of budesonide in AMS. The fact that GCs prevent AMS may suggest that AMS is in fact nothing else than a hypoxia-induced general inflammatory response. Also, more clinical trials comparing the benefits of NSAIDs to synthetic GCs in the prevention of AMS are recommended.

### How Does Hypoxia Cause Generalized Inflammation?

#### Physical Interaction Between NF-κB and HIF Proteins

**Figure 6:**
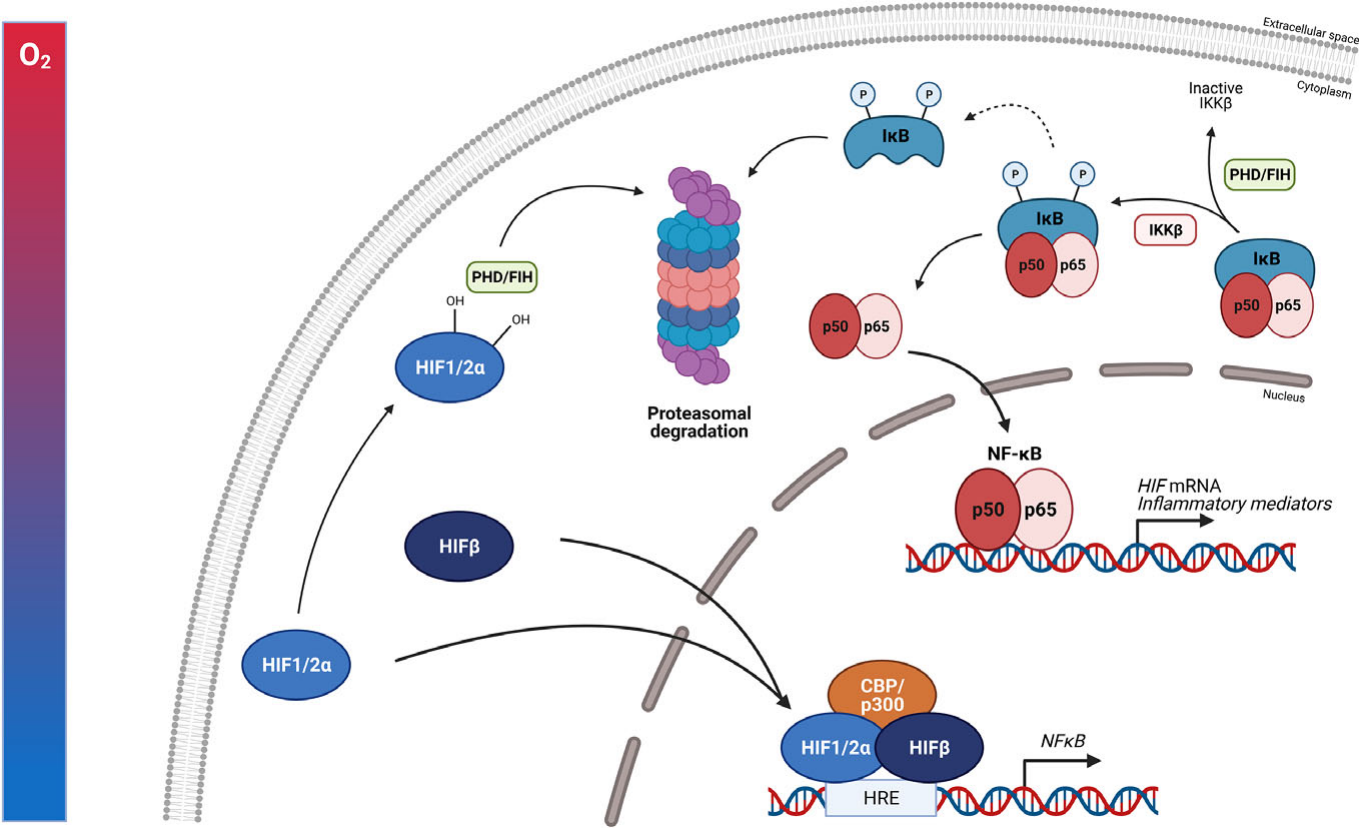
NF-κB protein regulation and the influence of HIF. The expression of NF-κB is induced by inflammatory stimuli and hypoxia. Inactive NF-κB is composed of a p50 and p65 subunit. The activation of this complex requires the degradation of inhibitory protein IκB. The phosphorylation of IκB by the β subunit of the IκB complex (IKKβ) leads to the degradation of IκB by the 26S proteasome. Once degraded, the NF-κB heterodimer can translocate to the nucleus where it can activate the transcription of inflammatory genes as well as HIF. PHDs and FIH can regulate the activation of NF-κB by controlling the IKKβ activity. Upon hypoxia, HIF heterodimers can translocate to the nucleus, bind to HREs and induce the transcription of numerous genes including NF-κB. Figures created with Biorender.

When humans ascent to high altitude and are exposed to hypoxic conditions, hypoxia itself is able to promote several TFs such as NF-κB thereby stimulating the production of pro-inflammatory cytokines like TNFα and IL-6 (121). NF-κB is a family of TFs composed of RelA (p65), RelB, c-Rel, NF-κB1 (p50/p105) and NF-κB2 (p52/p100). Different stimuli are able to activate these different subunits e.g. bacterial LPS, viral pathogens, cytokines and growth factors. NF-κB activation requires the degradation of the inhibitory proteins such as IκBs. Once they are phosphorylated by the IκB kinase (IKK) complex, IκBs are degraded by the proteasome and the NF-κB subunits can translocate to the nucleus to activate their target genes. One of these target genes is the HIFα subunit, thereby increasing its transcription (122). Several studies have reported the physical interaction between HIF and NF-κB. Hypoxia itself is also able to stimulate the activation of NF-κB. In the presence of hypoxia, PHD1 activity is inhibited thereby decreasing PHD-dependent hydroxylation of IKKβ, since IKKβ contains a conserved motif for PHD hydroxylation. This results in the phosphorylation of IKKβ and liberation of NF-κB from the cytoplasm (123). It is shown that HIF1α binds to RelA in EGF-induced cells (124). Also a novel and specific interaction between NF-κB essential modulator IKKγ (NEMO) and HIF2α has been reported by Bracken et al. This interaction enhances the transcriptional activity of HIF2α in normoxia in which NEMO promotes CBP/p300 recruitment to HIF2α (125). HIF1β is also able to interact physically with NF-κB. Namely, in CD30 stimulated cells, HIF1β interacts with RelB and p52 subunits thereby promoting NF-κB mediated transcription (126). Next to hypoxia, HIF is also induced by growth factors such as insulin-like growth factor 1 and platelet-derived growth factor, cytokines like TNFα and IL-1, and ROS, all of which are activators of NF-κB (**Figure 6**). This learns us that NF-κB functions as a direct modulator of HIF expression by regulating basal, TNFα and hypoxia-induced HIF expression (127–129). More elaborate studies will be necessary to determine whether the physical interaction between HIF and NF-κB is dependent on DNA-binding or is the result of protein-protein interactions. This is important, as more precise intervention strategies, involving GR or not, can then be developed.

#### Systemic Inflammation in Response to High Altitude

It has been shown that HIF1α also plays an important role in promoting the expression of NF-κB regulated inflammatory cytokines in macrophages after LPS (130). In humans ascending to high altitude, Hartmann et al. have reported elevated levels of the pro-inflammatory cytokine IL-6, the inflammatory marker IL-1 receptor antagonist (IL-1ra) and C-reactive protein (CRP) in the blood compared to normal levels. This demonstrates the presence of moderate systemic inflammation in response to high altitude (131). Also other studies have shown that exposure to hypoxia of rodents and humans causes increased vascular leakage and oxidative stress with elevated NF-κB expression in the lungs followed by significantly higher levels of pro-inflammatory cytokines IL-1, IL-6 and TNFα (132–134). Circulating chemokines known to recruit and activate leukocytes during inflammation such as macrophage inflammatory protein 1α (MIP-1α) and monocyte chemoattractant protein 1 (MCP-1) are also significantly upregulated in HAPE susceptible individuals (fast ascending mountaineers with previous episodes of HAPE) which is suggestive for chronic inflammation (135). DEX is able to block the production of MCP-1 generated by hypoxic alveolar macrophages to increase capillary permeability (136, 137). It also blocks the formation of migration inhibitory factor (MIF) (138). The high recurrence rate of HAPE susceptibility might point towards a genetic predisposition linked with PAH. For example, a missense mutation in the *JAK2* gene represents a good candidate gene for PAH and the possible development of HAPE upon ascending to high altitude. Also variants in the *CYP1B1* and *HRG* gene have been identified in HAPE susceptible mountaineers (139). Therefore, the identification of more candidate gene polymorphisms in the pathogenesis of HAPE in combination with its prevention and treatment will be of great importance in the better understanding of the disease and formulating new strategies to deal with it.

A recent prospective observational trial has shown that several pro-inflammatory cytokines and chemoattractant proteins are increased after 24h when non-acclimatized humans ascend to high altitude and can be associated with an increased LLS and the clinical symptoms for AMS. In blood samples of these individuals, levels of IL-1β, MCP-1 and its target protein VEGFA are increased at high altitude (140). When AMS further develops into HACE, both in humans and rats the high-altitude hypoxia causes an increase in circulating TNFα, IL-1β and IL-6 cytokines. Also the stress hormone CRH is significantly increased. In rats, this has been associated with the upregulation of pro-inflammatory cytokines which can be blocked with a CRH receptor type 1 (CRHR1) antagonist. Based on these findings, one could suggest that the hypoxia-activated CRH and its CRHR1 signalling might be important for the induction AMS and its pro-inflammatory response (141).

**Figure 7:**
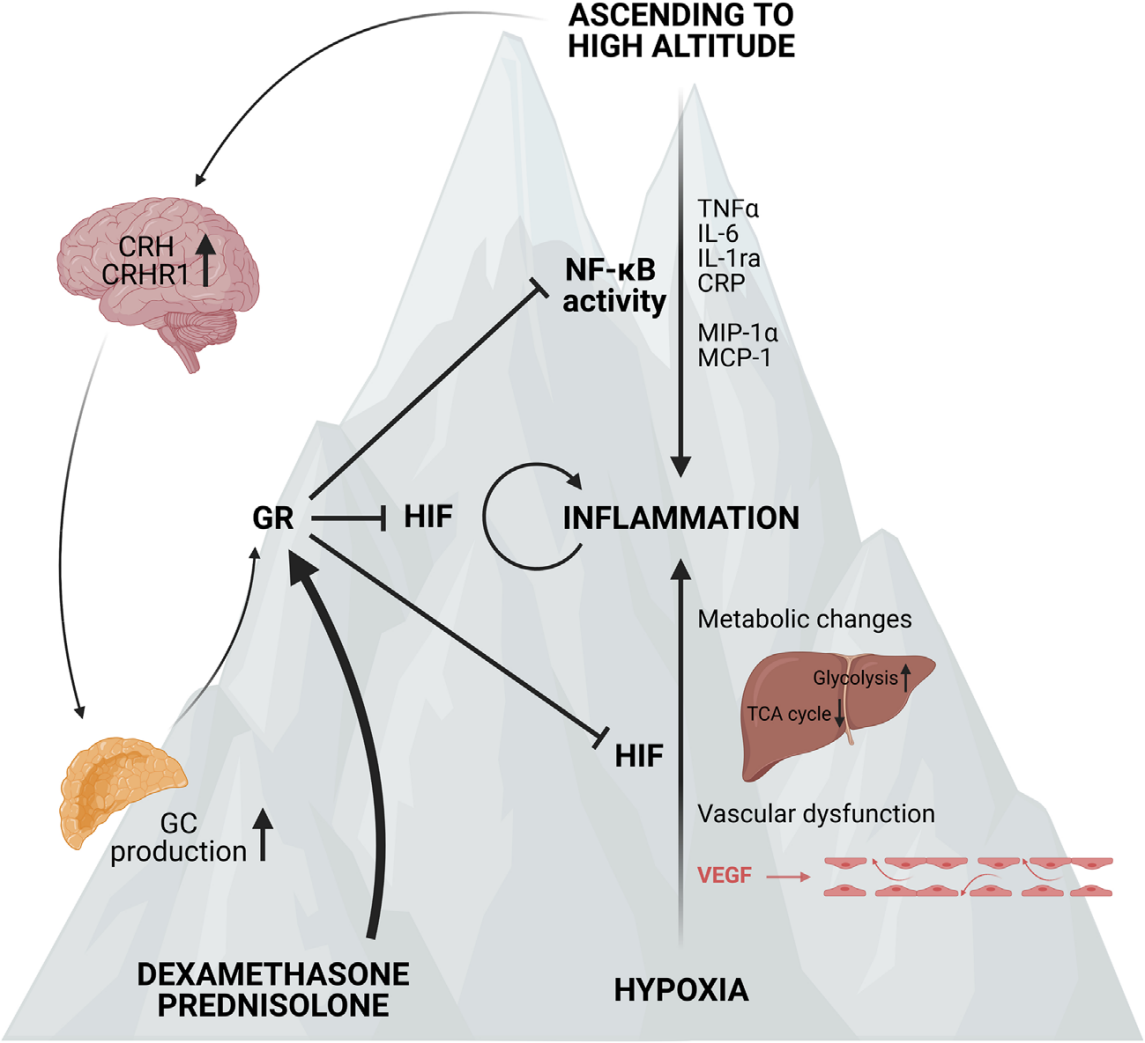
Acute mountain sickness (AMS) and the prophylactic effect of synthetic GCs. Ascending to high altitude can lead to the development of AMS. This is characterized by elevated NF-κB expression and higher levels of po-inflammatory markers such as TNFα, IL-6, IL-1ra, CRP and chemokines like MCP-1 and MIP-1α. Next to the inflammatory response, the expression of CRH and its receptor CRHR1 in the hypothalamus is also elevated, leading to increased GC production by the adrenal glands and stimulation of the GR. When ascending to high altitude, the hypoxic response is also characterized by metabolic changes and vascular dysfunction induced by the vascular endothelial growth factor (VEGF) contributing to the inflammatory response. When pre-treated with synthetic GCs such as dexamethasone and prednisolone, the GR is stimulated and the NF-κB and HIF mediated responses are inhibited and the symptoms of AMS are reduced. Figures created with Biorender.

In general, ascending to high altitude of non-acclimatized humans causes the development of AMS, which can further develop into HAPE or HACE. This is clearly associated with the induction of a pro-inflammatory response, most likely linked with a higher NF-κB activity and perhaps the involvement of CRH and the CRHR1 signalling. Small scale clinical trials suggest a prophylactic effect of acetazolamide and GCs such as DEX and prednisolone against AMS probably by reducing NF-κB activity and the production of pro-inflammatory cytokines (**Figure 7**). It would be of great interest to conduct large scale clinical trials with healthy individuals ascending to high altitude which could provide new insights into the body’s reaction to hypoxia. The exploration of possible mechanisms responsible for the adaptation to acute hypoxia might be of great added value to improve the understanding of causes and consequences of hypoxia in critical illness. Also the mechanism on how GR is able to inhibit HIF mediated effects in AMS, at the level of HIF itself or more downstream on HIF triggered inflammatory pathways might also be important in critically ill and septic patients and lead to the identification of new therapeutic targets.

## Oxygen Deprivation During Birth and the Role of GCs

Hypoxia during the period before and during parturition, and also after birth is one of the most common causes of neonatal morbidity followed by admission to the intensive care unit and neonatal mortality. Causes of foetal hypoxia might include unhealthy behaviour and the presence of chronic diseases in pregnant women (e.g. cardiovascular diseases, diabetes and anaemia). Also impairment of the foetal-placental barrier and exposure to harmful environments can lead to foetal hypoxia (142, 143). Postnatal hypoxia can appear from birth until days to weeks after parturition and is caused by a variety of cardiovascular and pulmonary disorders e.g. lack of lung development following preterm birth or patent ductus arteriosus in which the ductus arteriosus fails to close after birth (144, 145). Hypoxia in the premature neonate is generally associated with a harmful conditions requiring mechanical ventilation and oxygen therapy, GCs and other supplementary therapies (146, 147). The adaptation of neonates to hypoxia requires a coordinated physiological response, which includes an increase in the release of GCs, but not aldosterone, from the adrenal cortex. This implies a zone-specific adaptation of the adrenal glands to hypoxia (148). *In vitro* studies have shown that adrenal cells isolated from hypoxic neonatal rats display increased steroidogenesis due to hypoxia-induced changes to the steroidogenic enzyme activity rather than an alterations in the expression of steroidogenic enzymes (149). This is in contrast with adult rodents where hypoxia leads to a decreased expression of steroidogenic enzymes (150) and HIF1α is a central regulator of steroidogenesis (98).

### The Effect of Neonatal Hypoxia on the Activity of the HPA Axis

To gain further insights into the effect of neonatal hypoxia on the HPA axis, Raff et al. exposed suckling rats to hypoxia from birth until 5-7 days of age. This results into increased basal GC levels while no differences in endogenous plasma ACTH levels are detected (89, 90). The augmentation in steroidogenesis appears to be partly mediated by an increase in intracellular controllers of mitochondrial cholesterol transport, namely the steroidogenic acute regulatory (StAR) and peripheral-type benzodiazepine receptor (PBR) proteins (89). Since GCs induce a negative feedback loop at the level of the hypothalamus and pituitary, most likely when the hypothalamus is exposed to a direct stimulus e.g. ether vapours, this increased GC levels will inhibit the ACTH response to this stimulus (90). Also when perinatal hypoxia is induced in pregnant dams, maintaining them in a hypoxic environment during parturition and maintaining pups in hypoxia until 2 weeks of age, the GC response to an acute stressor in adult rats is significantly higher. This enhanced GC response is linked to higher CRH mRNA levels in the paraventricular nucleus (PVN) of the hypothalamic cells (151). The exposure of pregnant rats to hypoxia during the gestational period important for the maturation of the hippocampus induces long-term impairments in the GC system of the progeny. The exposure of pregnant females to adverse effects such as hypoxia causes an excess of GCs produced and a weakened negative feedback control of the HPA axis activity, resulting in higher baseline GC levels (152). This is associated with decreased nuclear GR protein levels (153) and reduced expression of GR dependent genes in the hippocampus of newborn rats. Additionally, the exposure to hypoxia during foetal development causes an age-related depletion of GR in the liver and is accompanied with a reduced efficacy of GC mediated processes such as the inability to maintain normal glucose levels by the liver (152).

### The Effects of GCs on Hypoxia-Ischemia Induced Neonatal Brain Injury

Next to GR, it is clear that HIF1α also plays a role in hypoxia-ischemia (HI) induced neonatal brain injury. The inhibition of HIF1α after a HI-induced injury results in neuroprotective effects by maintaining the blood-brain-barrier (BBB) integrity and reducing brain edema (154). On the contrary, when HIF1α is induced in a severe neonatal HI model, the BBB permeability is attenuated by the inhibition of VEGFA (91). It is clear that the exposure to perinatal and postnatal hypoxia causes a higher GC response, however a more detailed understanding of the mechanism and the timing of the HPA axis response to acute hypoxia remains to be further elucidated. Furthermore, the exposure to hypoxia during the perinatal period does have long-term pathophysiological effects that persist until adulthood. Next to that, a role of GR and HIF1α in HI-induced brain injury in neonates is obvious. It is likely that GR and HIF1α influence the adaptive response to a HI-induced insult thereby causing metabolic, apoptotic and inflammatory differences.

#### Neuroprotective Mechanisms of GCs

Perinatal hypoxia results in a significant increase in hypoxia-induced brain infarct size in neonates. The question remains whether treatment of neonatal HI-induced brain injury with exogenous GCs like DEX provide neuroprotective or neurotoxic effects. It is shown by Tuor et al. that DEX-mediated alterations in metabolism have a protective effect against neonatal HI-induced brain damage (92, 93). Additionally, the inflammatory response in HI injury is attenuated when neonates are treated with GCs by significantly reducing TNFα production (155). GCs also provide neuroprotective effects by inhibiting cleaved caspase-3. Hence, treatment of neonates with exogenous GCs affects downstream apoptotic pathways and decreased neuronal cell death in the brain injury. Furthermore, DEX also exerts neuroprotective effects by activating phosphorylated Akt, which is important in neuronal pro-survival signals (156). In general, the protective effects of DEX focuses more on the mechanisms that cause damage to neuronal cells rather than decreasing ROS (92, 157). When DEX is administered *via* an intracerebroventricular (ICV) injection prior to hypoxia-ischemia (HI), a concentration-dependent neuroprotective effect of DEX on HI-induced brain injury is present in which the VEGF pathway is partially involved (158). This suggests a direct, local neuroprotective effect of the activation of GR in the neonatal brain.

#### Neurotoxic Mechanisms of GCs

**Figure 8:**
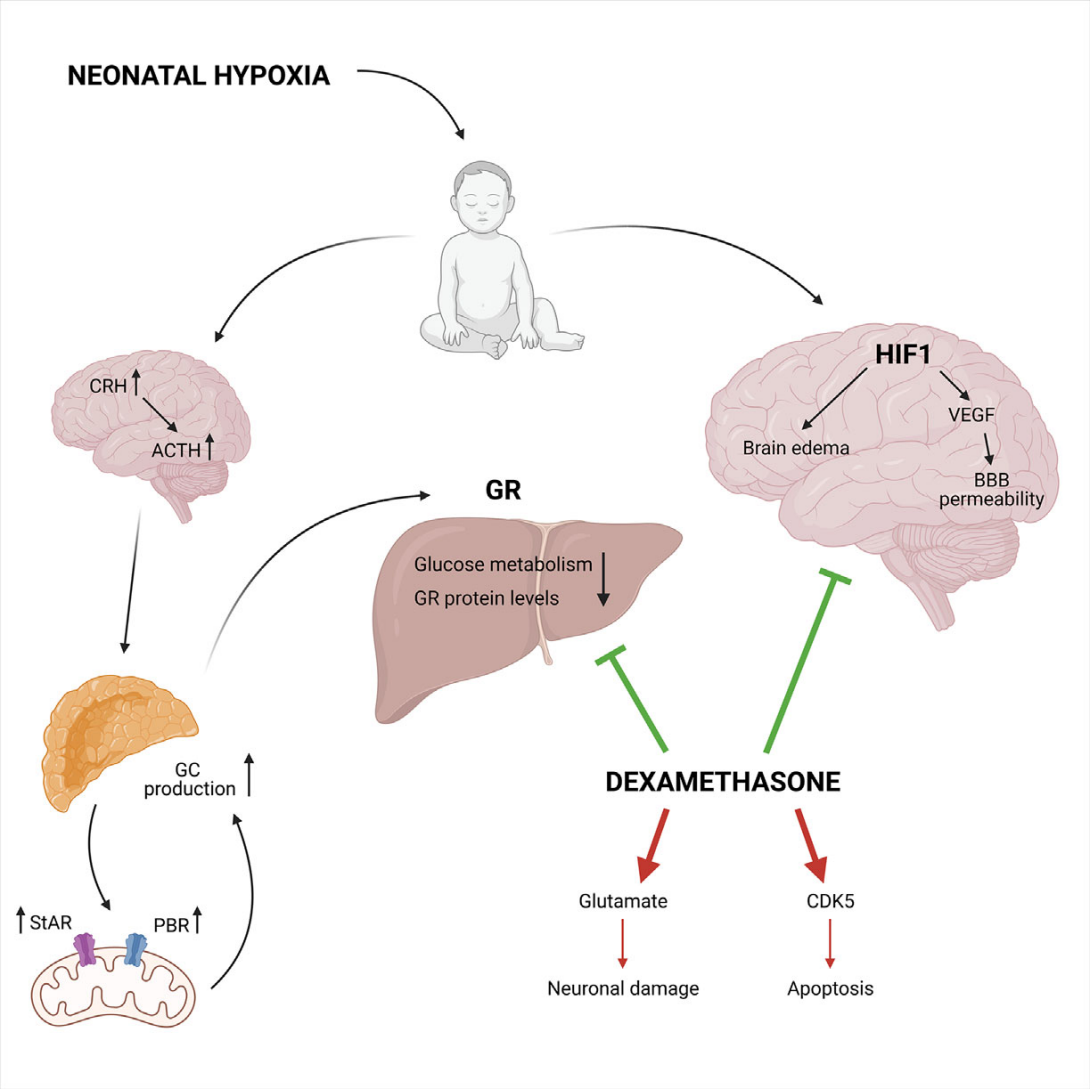
The neuroprotective and neurotoxic effects of synthetic GCs during neonatal hypoxia. Neonatal hypoxia activates the HPA axis leading to increased GC production, partly mediated by a higher StAR and PBR expression involved in the mitochondrial cholesterol transport. This leads to decreased GR protein levels and reduced glucose metabolism. On the other hand, HIF1 is involved in the hypoxia-ischemia induced brain injury *via* the induction of VEGF and increases blood-brain barrier permeability in combination with brain edema. Neuroprotective effects can be induced by DEX *via* the inhibition of HIF1 mediated brain injury and the inhibition of the GR mediated effects in the liver. Next to the neuroprotective effects, DEX also has neurotoxic effects. It can lead to increased glutamate levels and CDK5 expression causing neuronal damage and apoptosis, respectively. Figures created with Biorender.

However, when exposed to foetal hypoxia the neuroprotective effect of DEX in the neonatal brain is abrogated, most likely due to the downregulation in GR protein levels in the developing brain. This implies a role for GR in the increased vulnerability to HI-induced brain injury in the neonate due to foetal stress (153). The dosing and duration of GC treatment determines the neuroprotective or neurotoxic outcomes. For example, chronic exposure to GCs before HI-induced brain injury results in exacerbated neuronal cell death and white-matter injure (159). GCs also cause an accumulation of glutamate in the brain thereby inducing cytotoxicity and neuronal damage, which contributes to the detrimental effects in neonatal brain injury (160). Also, GCs enhance apoptotic cell death *via* overexpression of cyclin-dependent kinase 5 (CDK5), which is an important kinase for cell cycle regulation (**Figure 8**) (161).

In conclusion, evidence is provided that treatment of neonatal HI-induced brain injury with DEX can be either neuroprotective or neurotoxic (94, 95). In most cases, when DEX is administered systematically and chronically, neurotoxicity remains present, but when DEX is administered directly into the brain, it becomes neuroprotective. The ability of GCs to suppress neuroinflammation caused by hypoxia is thus dependent on dosing, timing and duration after the initial injury.

## Conclusion

The bidirectional crosstalk between GR and HIF is clearly highlighted based on the studies described above. *In vitro* studies have shown cell-type specific effects of exogenous GCs on hypoxia-dependent gene expression and HIF1α activity upon hypoxia, which is associated with effects on the nuclear translocation and DNA binding of HIF. Conversely, hypoxia is also able to exert an effect on the GR mRNA and protein levels by influencing GR nuclear translocation or due to the binding of HIF1 to HREs present at the *NR3C1* promotor. Only a few *in vivo* studies using zebrafish as a model organism have addressed the direct interaction between HIF and GR, thereby showing that HIF represses GR activity and its responsiveness to exogenous GCs. Moreover, HIF signalling reduces GC production by acting on POMC expression at the level of the hypothalamus. Further *in vivo* studies will be necessary to elucidate the precise crosstalk between these two complex signalling pathways.

**Figure 9:**
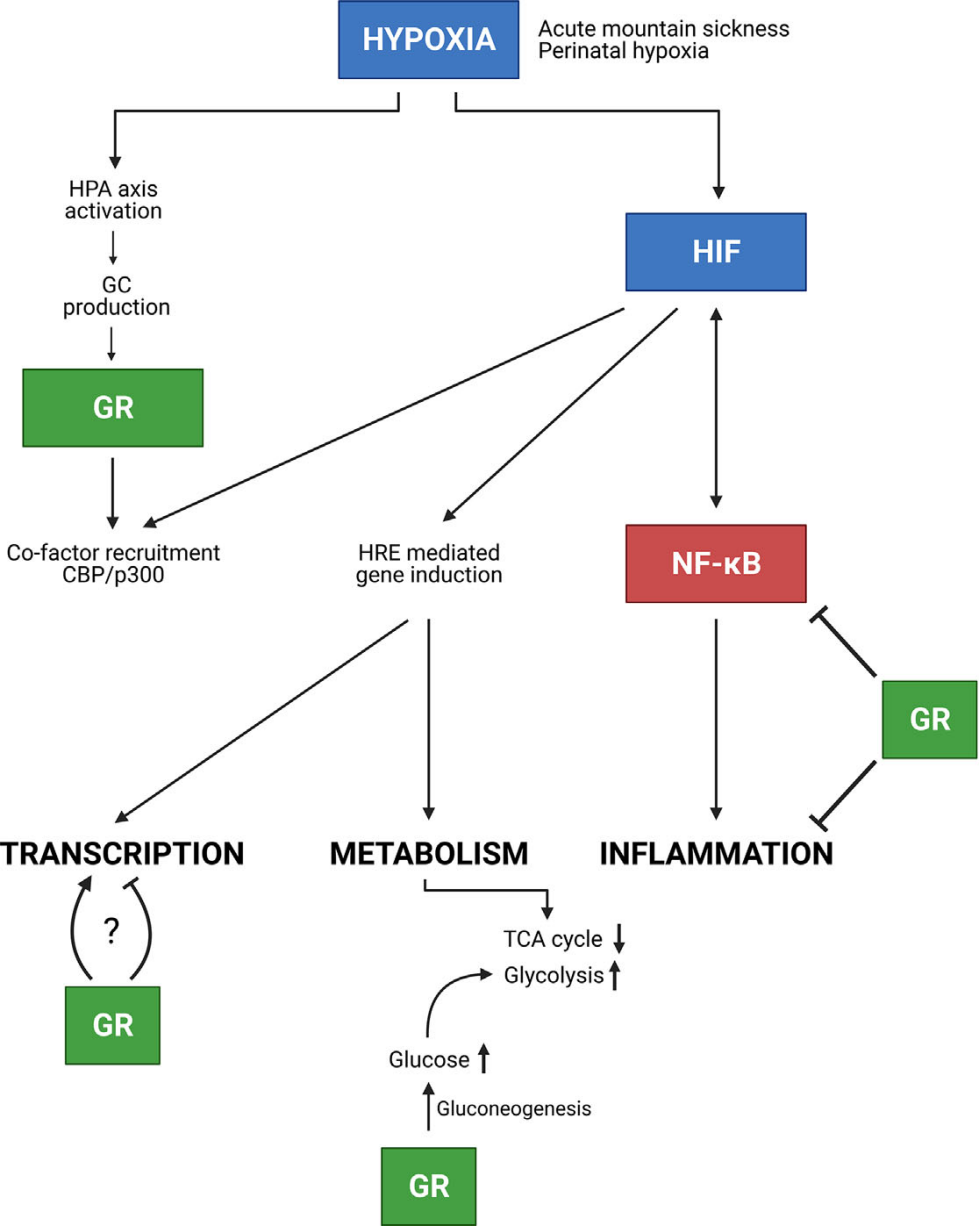
Conclusion. In the presence of hypoxia e.g. due to AMS or neonatal hypoxia, the HPA axis is activated thereby increasing GC production and GR mediated effects. Next to GR, HIF is involved in NF-κB inflammatory responses, in which these responses can be inhibited by GR. Furthermore, HIF mediated transcription is stimulated, but GR competes with HIF in the recruitment of co-factors e.g. CBP/p300. Whether GR stimulates or inhibits HIF mediated transcription requires further investigation. Finally, hypoxia also causes metabolic changes. The GR promotes gluconeogenesis thereby increasing glucose levels which will be used during glycolysis for appropriate energy levels in hypoxic conditions. Figures created with Biorender.

GCs are known to be strong anti-inflammatory mediators. However, in this review, it is shown that hypoxia is able to affect these anti-inflammatory effects by influencing the DEX mediated suppression of pro-inflammatory cytokine production. On the contrary, hypoxia leads to higher GILZ levels in macrophages when exposed to an inflammatory stimulus to prevent overactivation of immune cells and overproduction of pro-inflammatory cytokines. This teaches us that a balance between GR and HIF mediated signalling is required upon inflammatory conditions and that it will be necessary to further investigate the interplay between HIF and GR in disease models such as IMH, PAH and sepsis. Also the link with NF-κB signalling is of utmost importance (**Figure 9**). The exploration of possible mechanisms responsible for the adaptation to acute hypoxia might be of great added value to improve the understanding of causes and consequences of hypoxia in critical illness. Also the mechanism on how GR is able to inhibit HIF mediated effects in AMS, at the level of HIF itself or more downstream on HIF triggered inflammatory pathways might also be important in critically ill and septic patients and lead to the identification of new therapeutic targets.

Since hypoxia is one of the most common causes of morbidity and mortality in neonates, a more complete understanding of what is the mechanism behind these hypoxic conditions in neonates will be of great added value. It is necessary to find the ideal dose, timing and during of GC treatment so that neurotoxic effects of GCs can be eliminated. Considering the importance of HIF1α during neural development, it could be relevant to further elucidate the use of PHD inhibitors or HIF stabilizers during neonatal hypoxia.

Overall, a clear interaction between HIF, GR and NF-κB is shown both *in vitro* and *in vivo*. It will be of importance to identify the role of specific HIF isoforms in several diseases and how this is connected with GR and/or NF-κB. Based on these results, specific targeting of certain HIF isoforms in inflammatory disease models could lead to the identification of new therapeutic targets.

## Author Contributions

TV wrote the draft, and RB and CL supervised, corrected, and finalized the paper. All authors contributed to the article and approved the submitted version.

## Funding

Research in the lab of CL and RB is supported by the Research Council of Ghent University (GOA Program), the Research Foundation Flanders (FWO-Vlaanderen), the FWO Hercules program and Flanders Institute for Biotechnology (VIB).

## Conflict of Interest

The authors declare that the research was conducted in the absence of any commercial or financial relationships that could be construed as a potential conflict of interest.
